# Supplementary material for: Asynchronous evolution of interdependent nest characters across the avian phylogeny
Source: Nat Commun. 2018 May 14;9:1863. doi: 10.1038/s41467-018-04265-x (PMC5951845; doi:10.1038/s41467-018-04265-x)
Supplement: Supplementary file 1 — Supplementary Information [file 41467_2018_4265_MOESM1_ESM.pdf]

Supplementary Information

Asynchronous evolution of interdependent nest characters across the avian phylogeny

Fang et al.

Supplementary Table 1. Results of conventional and EM-based Mantel tests for the three nest characters based on the "effective" datasets for all bird families (Bird) and all passerine families (Passerine).

	Structure		Site		Attachment	
	Bird	Passerine	Bird	Passerine	Bird	Passerine
Mantel r statistic	0.44	0.18	0.33	-0.01	-0.16	0.16
Convention (P value)[#]	<0.01	<0.01	<0.01	0.43	<0.01	0.01
EM (P value)[#]	0.02	0.13	0.05	0.31	0.01	0.13

[#] The null hypothesis for all tests is r <= 0 except for attachment in the all bird dataset and site in the all passerine dataset, for which the null hypothesis is r >= 0

Supplementary Table 2. Results of conventional and EM-based Mantel tests for the three nest characters based on the "all" datasets for all bird families (Bird) and all passerine families (Passerine).

	Structure		Site		Attachment	
	Bird	Passerine	Bird	Passerine	Bird	Passerine
Mantel r statistic	0.44	0.21	0.28	<0.01	-0.17	0.19
Convention (P value)[#]	<0.01	<0.01	<0.01	0.48	<0.01	<0.01
EM (P value)[#]	0.02	0.11	0.06	0.58	<0.01	0.11

[#] The null hypothesis for all tests is r <= 0 except for attachment in the all bird dataset, for which the null hypothesis is r >= 0

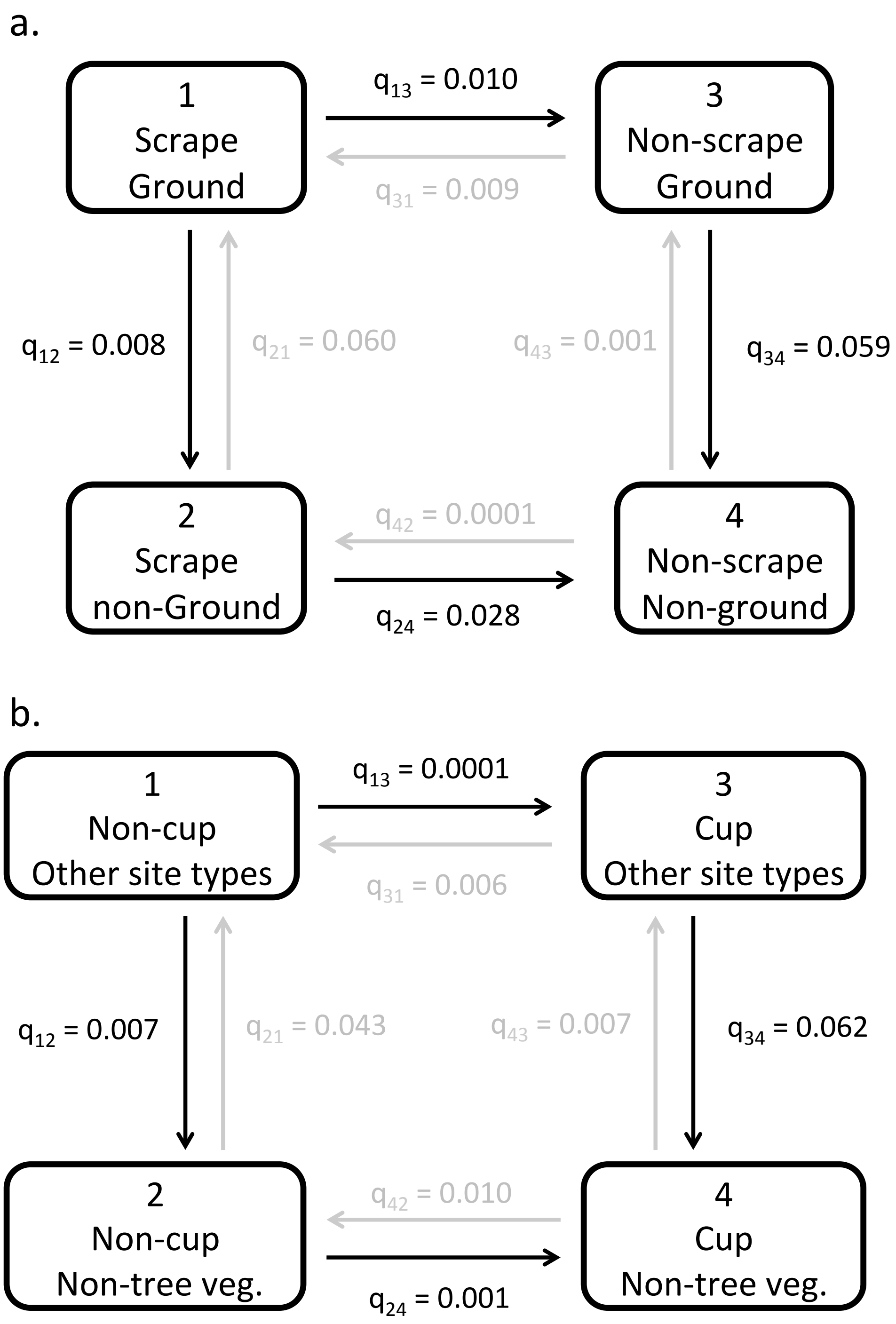
a.
1
Scrape
Ground
q13 = 0.010
q31 = 0.009
3
Non-scrape
Ground
q12 = 0.008
q21 = 0.060
q43 = 0.001
q34 = 0.059
2
Scrape
non-Ground
q42 = 0.0001
q24 = 0.028
4
Non-scrape
Non-ground
b.
1
Non-cup
Other site types
q13 = 0.0001
q31 = 0.006
3
Cup
Other site types
q12 = 0.007
q21 = 0.043
q43 = 0.007
q34 = 0.062
2
Non-cup
Non-tree veg.
q42 = 0.010
q24 = 0.001
4
Cup
Non-tree veg.

c.

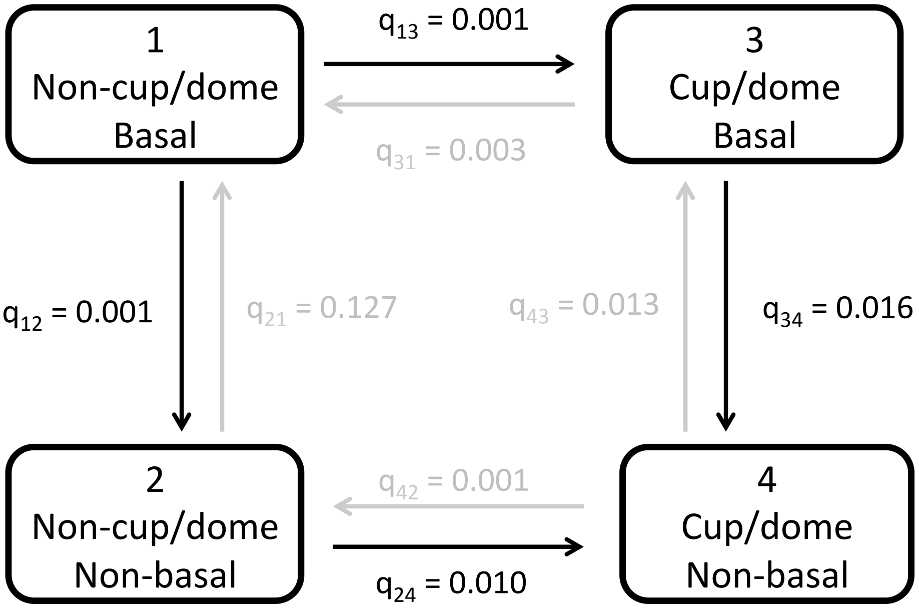

Supplementary Figure 1. Evolutionary transition rates between nest character states under dependent models. (a) The transition from scrape nests on the ground to nests with other structure and site types is more likely via a non-scrape structure intermediate (q13 + q34 = 0.069) than a non-ground intermediate (q12 + q24 = 0.036). Furthermore, the transition rate from ground to other site types is higher in non-scrape nesters (q34 = 0.059) than scrape nesters (q12 = 0.008). (b) The transition pathway to cup nests on non-tree vegetation site from other structure and site types is more likely via a cup nest intermediate (q13 + q34 = 0.062) than a non-tree vegetation intermediate (q12 + q24 = 0.008). The transition rate to non-tree vegetation from other site types is higher in cup nesters (q34 = 0.062) than birds building other structure types (q12 = 0.007). (c) The transition pathway to cup/domed nests with non-basal attachment from other structure types with basal attachment is more likely via a cup/domed nest intermediate (q13 + q34 = 0.017) than a non-basal attachment intermediate (q12 + q24 = 0.008). The transition to non-basal attachment is more likely to occur in cup/domed nesters (q34 = 0.016) than birds building other structure types (q12 = 0.001).

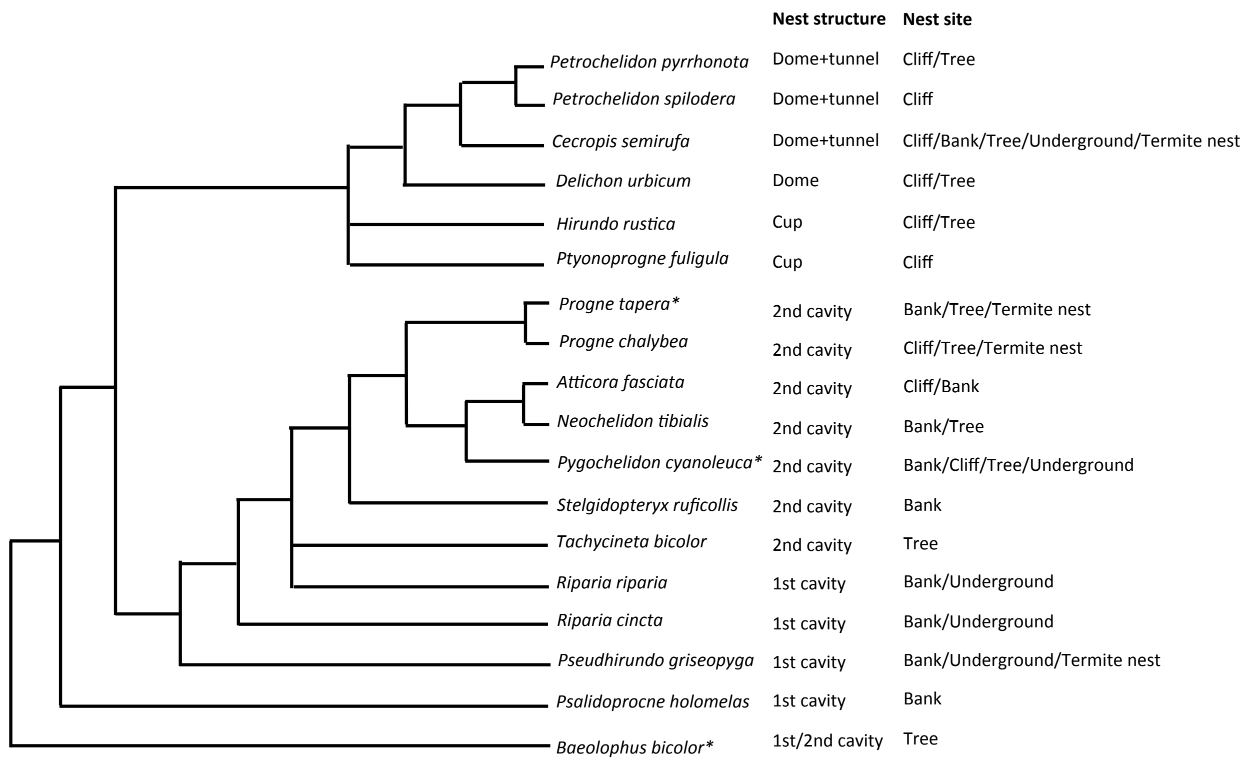

Supplementary Figure 2. Distribution of the character states of nest structure (data from ref. 2) and nest site (data from this study) for swallows in the their phylogeny (Modified from ref. 2). * indicates species with recently revised names compared to that in ref. 2: *Progne tapera = Phaeprogne tapera; Pygochelidon cyanoleuca = Notiochelidon cyanoleuca; Baeolophus bicolor = Parus bicolor.*

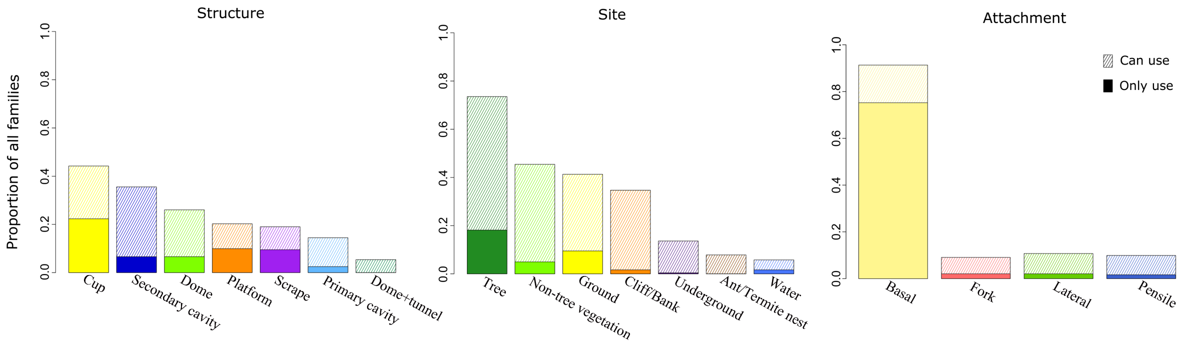

Supplementary Figure 3. Same as Fig. 1, but based on all character states mentioned on the family summary pages in the HBW[1] (Supplementary Data 2).

a

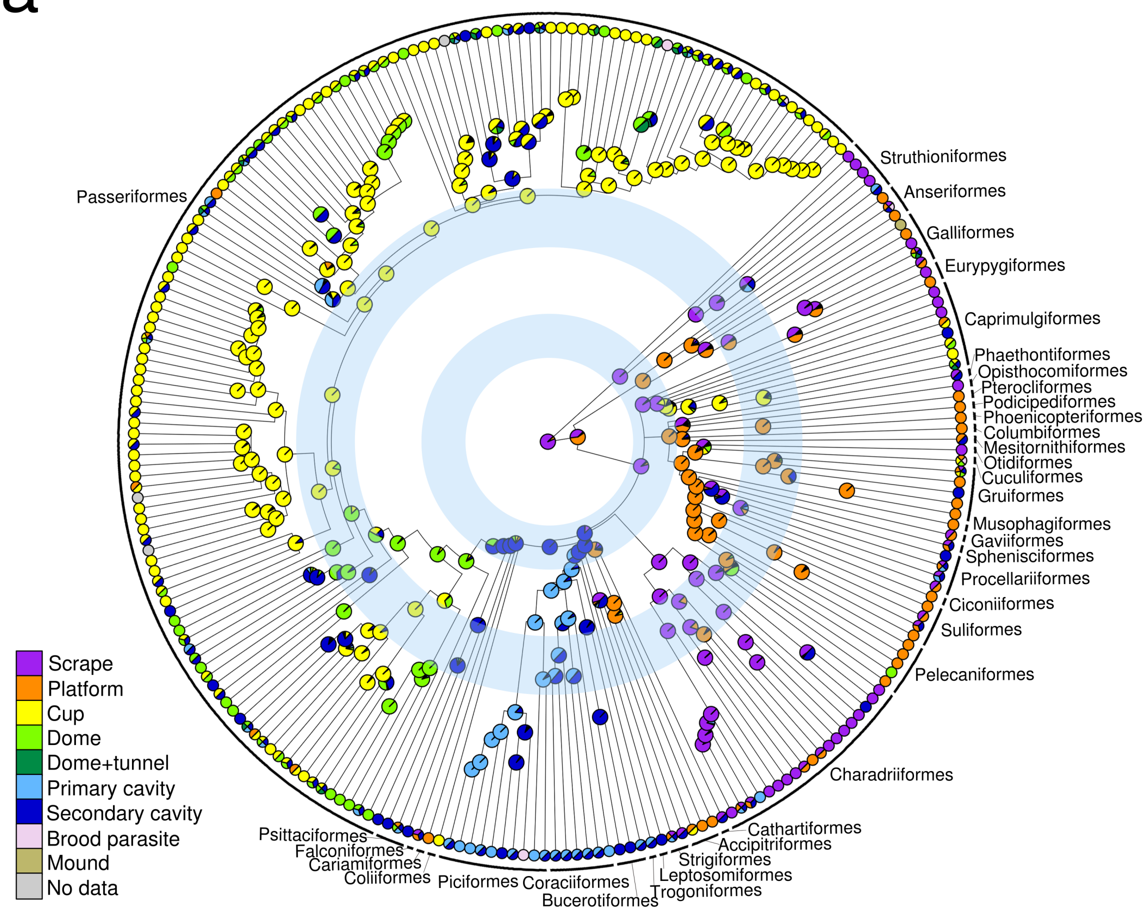
Struthioniformes
Anseriformes
Galliformes
Eurypygiformes
Caprimulgiformes
Phaethontiformes
Opisthocomiformes
Pterocliformes
Podicipediformes
Phoenicopteriformes
Columbiformes
Mesitornithiformes
Otidiformes
Cuculiformes
Gruiformes
Musophagiformes
Gaviiformes
Sphenisciformes
Procellariiformes
Ciconiiformes
Suliformes
Pelecaniformes
Charadriiformes
Cathartiformes
Accipitriformes
Strigiformes
Leptosomiformes
Trogoniformes
Bucerotiformes
Coraciiformes
Piciformes
Coliiformes
Cariamiformes
Falconiformes
Psittaciformes
Passeriformes
Scrape
Platform
Cup
Dome
Dome+tunnel
Primary cavity
Secondary cavity
Brood parasite
Mound
No data

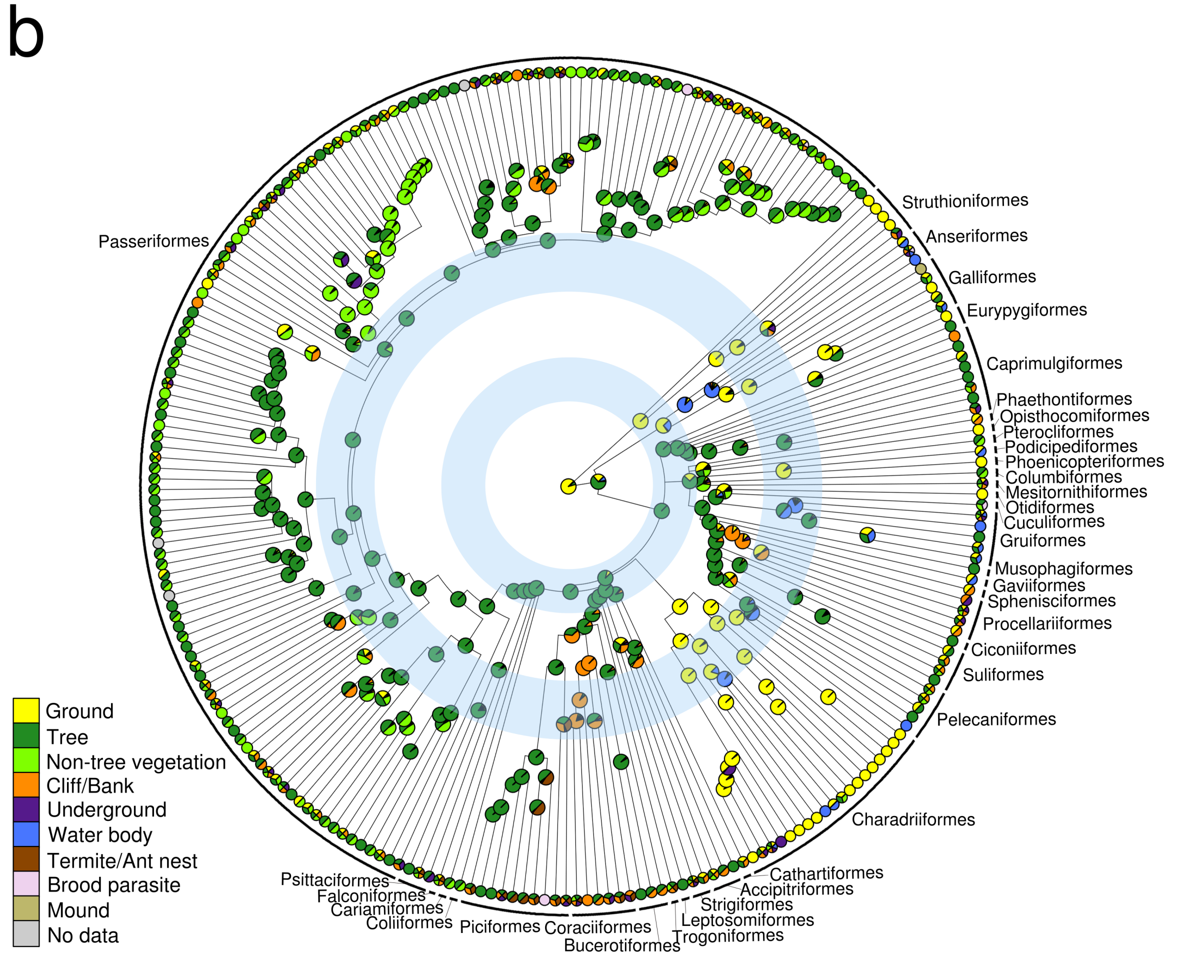
b
Passeriformes
Struthioniformes
Anseriformes
Galliformes
Eurypygiformes
Caprimulgiformes
Phaethontiformes
Opisthocomiformes
Pterocliformes
Podicipediformes
Phoenicopteriformes
Columbiformes
Mesitornithiformes
Otidiformes
Cuculiformes
Gruiformes
Musophagiformes
Gaviiformes
Sphenisciformes
Procellariiformes
Ciconiiformes
Suliformes
Pelecaniformes
Charadriiformes
Cathartiformes
Accipitriformes
Strigiformes
Leptosomiformes
Trogoniformes
Bucerotiformes
Coraciiformes
Piciformes
Coliiformes
Cariamiformes
Falconiformes
Psittaciformes
Ground
Tree
Non-tree vegetation
Cliff/Bank
Underground
Water body
Termite/Ant nest
Brood parasite
Mound
No data

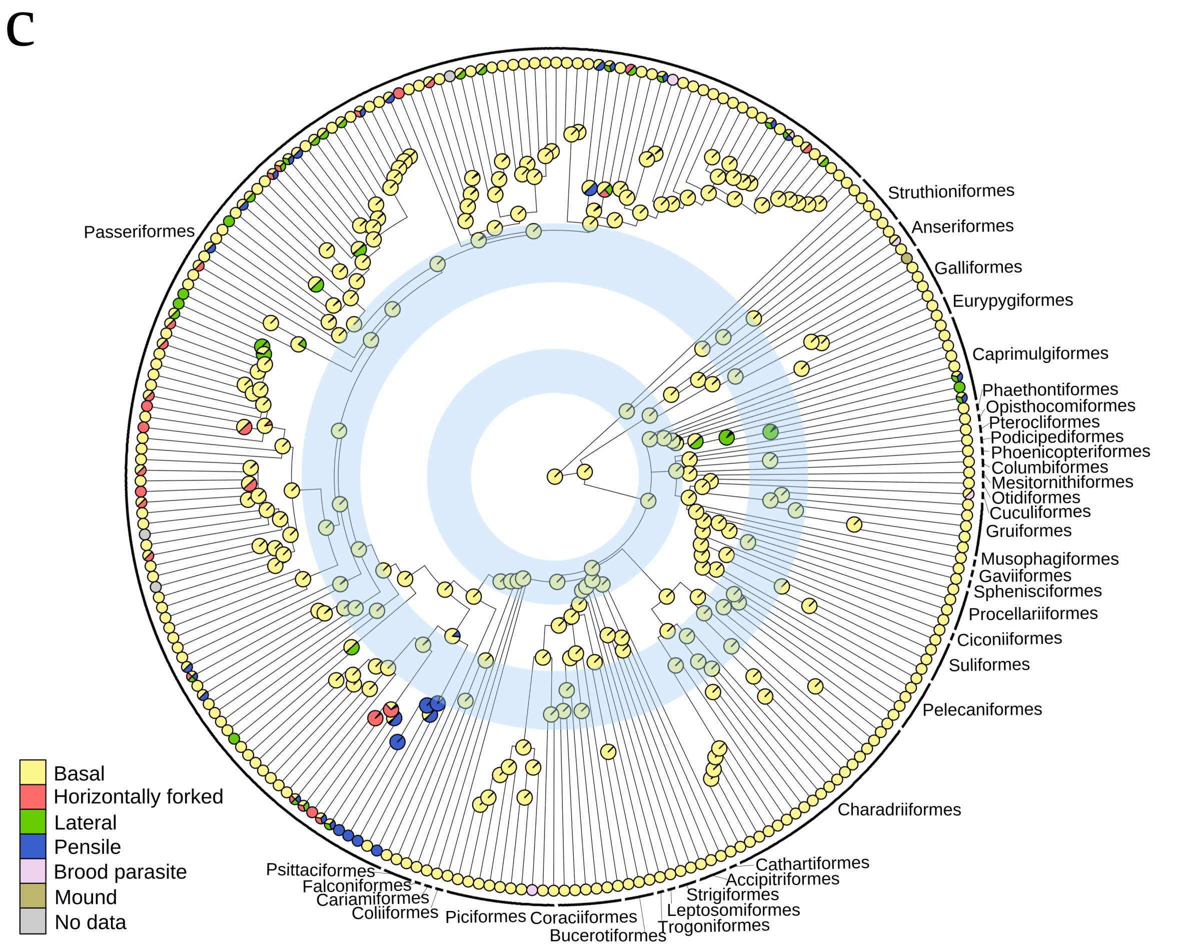

Supplementary Figure 4. Same as Fig. 2, but based on all character states mentioned on the family summary pages in the HBW[1] (Supplementary Data 2).

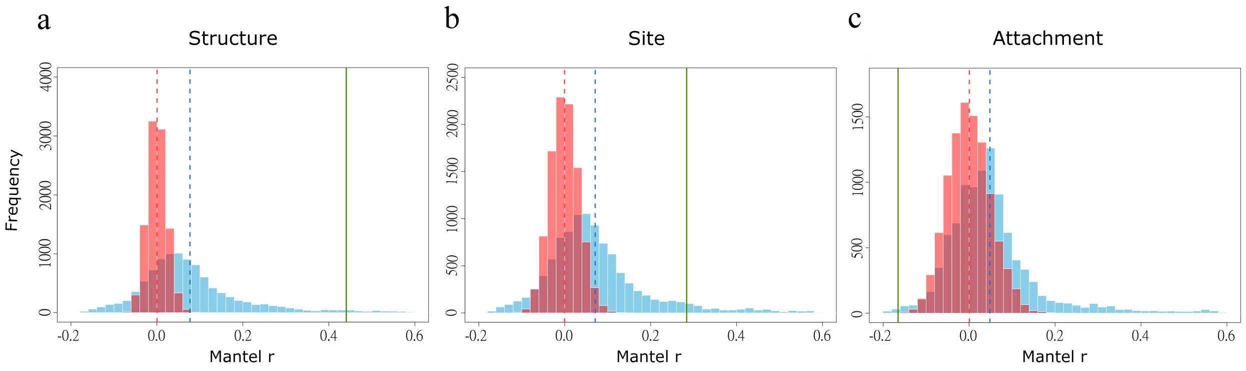

Supplementary Figure 5. Same as Fig. 3, but based on all character states mentioned on the family summary pages in the HBW[1] (Supplementary Data 2).

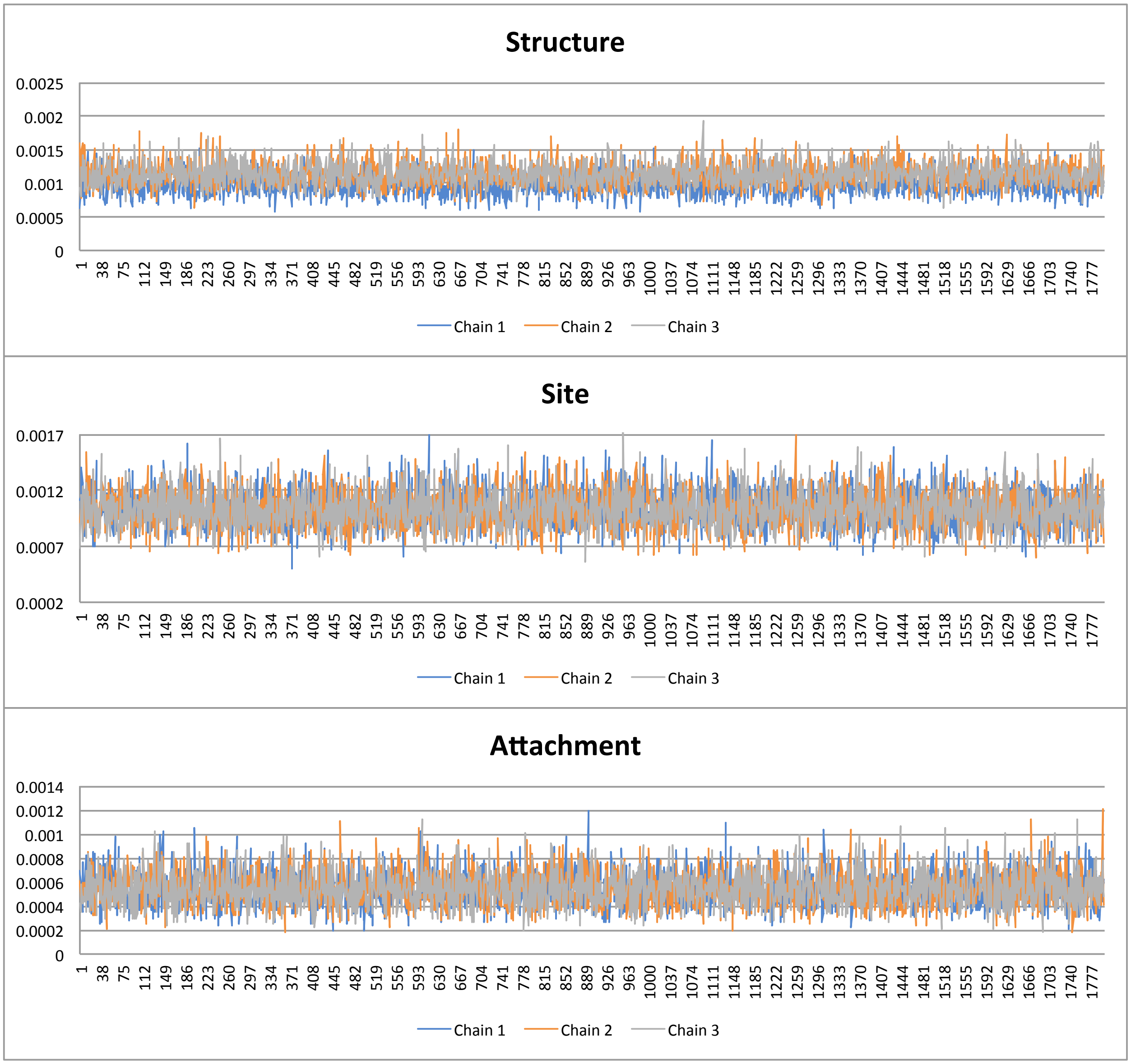

Supplementary Figure 6. Trend lines of three independent MCMC runs in BayesTraits analyses for nest structure, site and attachment. The analyses are based on the equal-substitution-rate model.

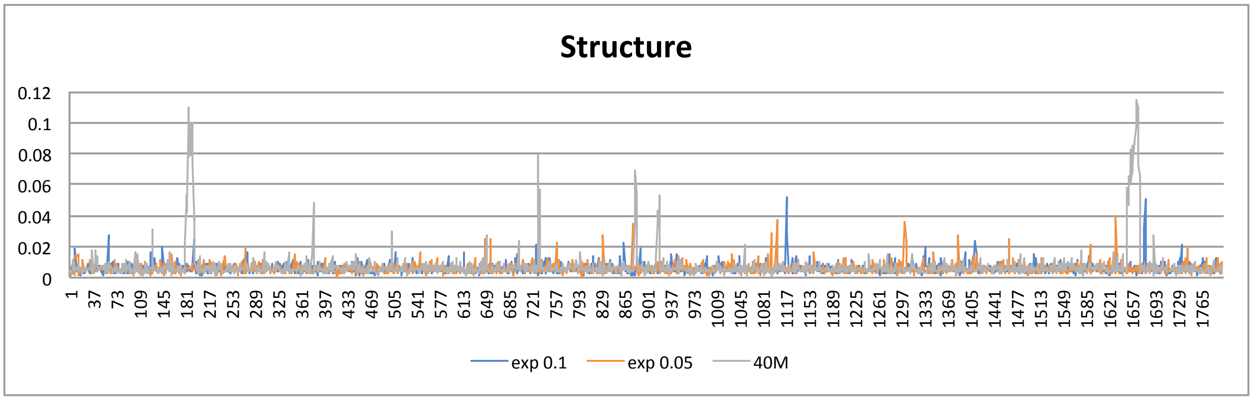

Supplementary Figure 7. Trend lines of MCMC runs in BayesTraits analyses for nest structure with two different priors (exp 0.1: exponential prior with a mean of 0.1; exp 0.05: exponential prior with a mean of 0.05) and with a longer chain (40M: 40 million iterations). The analyses are based on the different-substitution-rate model.

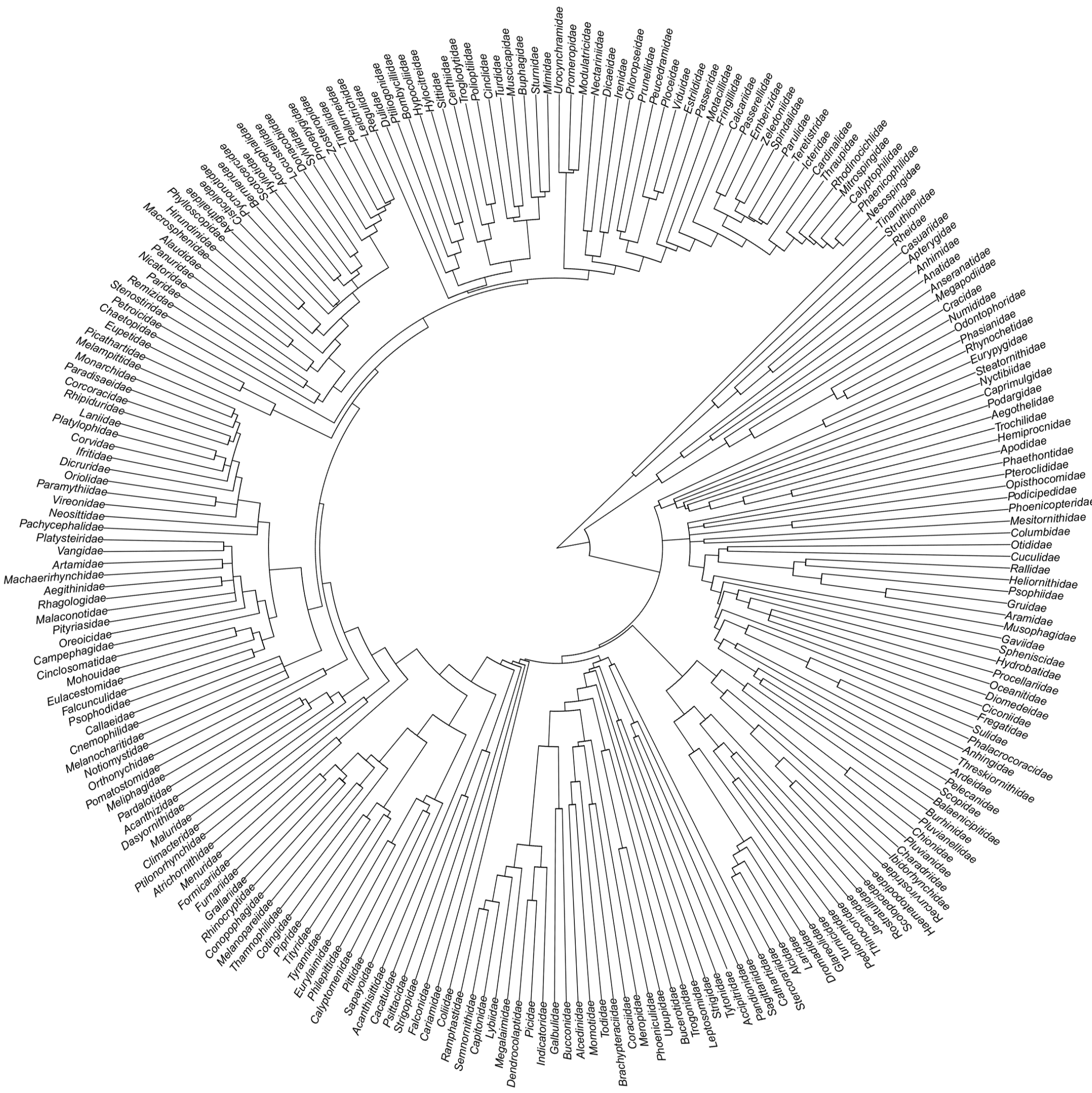

Supplementary Figure 8. A majority-rule consensus tree generated from the 1,000 family trees extracted from BirdTree.org based on the Hackett constraint. The names of 242 avian families are labeled on the terminal branches. The consensus tree is used to summarize and visualize the distribution of current and ancestral character states in Fig. 2 and Supplementary Fig. 4.

Supplementary Note 1

Methods and Results of MuSSE analyses

Methods

We used the MuSSE (Multi-State Speciation and Extinction) function of the diversitree[3] package in R to test the effect of nest structure types on the speciation rates (lambda) of lineages. We separated the nest structure types to three categories: (1) scrape, (2) platform/cavity and (3) cup/dome. We compared the 'state-dependent' model, in which speciation and extinction rates were impacted by the states of tested characters, with the 'state-independent' model, in which speciation and extinction rates were independent from character states[3]. If the former was significantly better supported than the latter, we concluded that the character states affected the speciation rates of lineages and then estimated the speciation rates of lineages associated with different states.

The MuSSE analyses were conducted based on the consensus tree generated from the 1,000 family trees, and each run of the MCMC chain contained 20,000 generations with the first 2,000 generations discarded as burn-in. We set an exponential prior with a mean of 0.1 for the estimated parameters. Three separate runs were conducted for each analysis to ensure convergence of parameter estimates. The reported parameters were averaged across the three runs. We used Tracer v1.6 (http://beast.bio.ed.ac.uk/Tracer) to assess runs for adequate mixing and to ensure properly high effective sample sizes (ESS > 1,500) for posterior probability distribution. We used Bayes Factors estimated from the harmonic means of log likelihoods to determine the level of support for 'state-dependent' models versus 'state-independent' models. A value of Bayes Factors larger than 5 indicated strong support for the 'state-dependent' model.

Results & Discussion

The 'state-dependent' model was strongly supported (Bayes Factor = 88.8), and thus we concluded that the nest structure types affected the speciation rates of avian lineages. The average speciation rate in avian lineages with scrape nests (lambda1 = 0.0607) was higher than that of cup/dome nests (lambda3 = 0.0309), followed by that of platform/cavity nests (lambda2 = 0.0001). However, the results revealed little information about whether the platform/cavity and cup/dome nests led to bursts of new species (or families) at the period of time when these structure types evolved. That is because the MuSSE estimates the speciation rates averaged across the evolution history (i.e., the whole phylogeny) rather than those at particular time points. Thus, we cannot use the approach to assess the effect of new nest structure types on the adaptive radiations in birds.

Supplementary References

1. de Hoyo, J., Elliot, A., Sargatal, J., Christie, D. A., de Juana, E. *Handbook of birds of the world alive*. Spain: Lynx Editions. See http://www.hbw.com/ (accessed October 2017)
2. Winkler, D. W. & Sheldon, F. H. Evolution of nest construction in swallows (Hirundinidae): a molecular phylogenetic perspective. *Proc. Natl. Acad. Sci. U.S.A.* **90,** 5705-5707 (1993).
3. FitzJohn, R. G. Diversitree: comparative phylogenetic analyses of diversification in R. *Methods Ecol. Evol*. **3,** 1084-1092 (2012).
