# Supplementary material for: Asynchronous evolution of interdependent nest characters across the avian phylogeny
Source: Nat Commun. 2018 May 14;9:1863. doi: 10.1038/s41467-018-04265-x (PMC5951845; doi:10.1038/s41467-018-04265-x)
Supplement: Supplementary file 3 — Description of Additional Supplementary Files [file 41467_2018_4265_MOESM3_ESM.pdf]

Description of Additional Supplementary Files

File Name: Supplementary Data 1
Description: Nest character states of all bird families. A character state is included in a family if more than 10% of the species in that family have that particular state (i.e., the "effective" dataset).

File Name: Supplementary Data 2
Description: Nest character states of all bird families. All character states mentioned on the family summary pages in the *Handbook of Birds of the World Alive* (HBW; http://www.hbw.com/) are included (i.e., the "all" dataset).
